# Application of plasma alternative to serum for measuring leucine-rich α2-glycoprotein as a biomarker of inflammatory bowel disease

**DOI:** 10.1371/journal.pone.0286415

**Published:** 2023-06-23

**Authors:** Tadashi Ichimiya, Tomoe Kazama, Keisuke Ishigami, Yoshihiro Yokoyama, Yuki Hayashi, Satoshi Takahashi, Takao Itoi, Hiroshi Nakase

**Affiliations:** 1 Department of Gastroenterology and Hepatology, Sapporo Medical University School of Medicine, Sapporo, Hokkaido, Japan; 2 Department of Infection Control and Laboratory Medicine, Sapporo Medical University School of Medicine, Sapporo, Hokkaido, Japan; 3 Department of Gastroenterology and Hepatology, Tokyo Medical University, Tokyo, Japan; Kurume University School of Medicine, JAPAN

## Abstract

**Background:**

Inflammatory bowel disease (IBD) is a chronic intestinal disorder characterized by recurrent flare-ups and remission. Leucine-rich α2-glycoprotein (LRG) has been developed as a new serum biomarker of disease activity in patients with IBD. However, there have been no reports on whether plasma LRG can be used as an alternative to serum LRG. Therefore, in this retrospective study, we evaluated the usefulness of plasma LRG compared to serum LRG.

**Methods:**

We conducted a single-center retrospective observational study. A total of 108 IBD patients (ulcerative colitis [UC], 56; Crohn’s disease [CD], 52) who received treatment at Sapporo Medical University Hospital between August 2020 and September 2021 were enrolled. Serum and plasma LRG levels were measured using the NANOPIA LRG kit. Disease activity was assessed using the Crohn’s Disease Activity Index (CDAI) for CD and partial Mayo (pMayo) score for UC. Endoscopic activity was evaluated using the Mayo Endoscopic Subscore (MES) and Ulcerative Colitis Endoscopic Index of Severity (UCEIS) in patients with UC and the Simple Endoscopic Score for Crohn’s Disease (SES-CD) score in patients with CD.

**Results:**

Serum LRG levels significantly correlated with plasma LRG levels (*r* = 0.990, *p*<0.0001). Plasma LRG levels were significantly associated with SES-CD (*r* = 0.992, *p*<0.0001), indicating that plasma LRG levels may predict endoscopic activity in CD. In UC patients, the cutoff values of plasma LRG for remission were 12.7 μg/mL for MES ≤1 and 10.0 μg/mL for UCEIS of = 0.

**Conclusion:**

The present study showed that plasma LRG levels correlate well with serum LRG levels. Therefore, plasma LRG can be clinically applied as a biomarker for assessing endoscopic disease activity in patients with IBD.

## Introduction

Inflammatory bowel disease (IBD) is an intractable chronic disease characterized by repeated flare-ups and remissions. The pathogenesis of IBD is thought to be complicated by various factors, including genetic factors, changes in intestinal microflora, and dietary history. However, the full extent of the disease is yet to be elucidated. According to the strict definition, IBD includes ulcerative colitis (UC) and Crohn’s disease (CD), and inflammatory biomarkers are very useful in accurately determining disease activity in both diseases. C-reactive protein (CRP), which is produced by tissue destruction due to inflammation, has long been regarded as an important inflammatory marker of disease activity in IBD [1, 2]. However, we often experience CRP-negative cases in clinical practice that are inconsistent with disease activity, and CRP does not necessarily correlate with disease activity in patients with CD [3]. CRP level alone may not be sufficient to adequately assess disease activity in patients with IBD. Leucine-rich α2 glycoprotein (LRG) has recently been developed as a new serum biomarker [4]. LRG is expressed not only in hepatocytes but also in neutrophils, macrophages, and intestinal epithelial cells [5]. Previous reports have demonstrated that serum LRG is a useful biomarker for predicting disease activity and mucosal inflammation in CD and UC [6, 7]. It is well known that the measurement time for plasma is at least 30 minutes shorter than that for serum. However, there are no reports on the clinical application of plasma LRG as an alternative to serum LRG. This study aimed to compare the correlation between plasma LRG and serum LRG, and investigate the relationship between plasma LRG and clinical or endoscopic disease activity in patients with UC and CD.

## Materials and methods

### Study design and participants

We conducted a single-center, retrospective, observational study at Sapporo Medical University Hospital. We enrolled patients with UC and CD who received treatment between August 2020 and September 2021. All patients were diagnosed with UC or CD based on these criteria [8].

Blood samples were collected from 56 patients with UC and 52 patients with CD at different times during the observational period. Based on the electronic medical records of the patients, background factors, disease activity, blood test results, and endoscopic findings were assessed.

### Assessment of LRG and disease activity

Serum LRG and plasma LRG levels were measured using the NANOPIA LRG kit based on the latex turbidimetric method (Sekisui Medical, Tokyo, Japan). Plasma and serum LRG measurements were performed on the same day.

Disease activity was assessed using the partial Mayo (pMayo) score for UC and the Crohn’s Disease Activity Index (CDAI) for CD. Endoscopic disease activity was assessed within one week before and after LRG measurements in both patients with UC and CD. Endoscopic activity was evaluated using the Mayo Endoscopic Subscore (MES) [9, 10] and Ulcerative Colitis Endoscopic Index of Severity (UCEIS) [11] in patients with UC and the Simple Endoscopic Score for Crohn’s Disease (SES-CD) score [12] in patients with CD. Endoscopic remission was defined as an MES of ≤1 [13] or UCEIS of = 0 [14] in UC and SES-CD of <4 in CD. Negative CRP level was defined as < 0.3 mg/dL.

### Statistical analysis

All statistical analyses were performed using JMP Pro (version 16.2.0, SAS Institute, Cary, NC, USA). Spearman’s correlation coefficient or Pearson’s correlation coefficient was used to analyze the association between plasma LRG, disease activity, and other biomarkers. In both analyses, *p*-values < 0.05 were considered statistically significant. Receiver operating characteristic (ROC) curve analysis was performed to evaluate diagnostic accuracy. The cutoff values were determined using the Youden index. A 95% confidence interval was obtained.

### Ethical consideration

The informed consent requirement has been waived due to the retrospective nature of this study. Instead, information about the study was posted on the hospital website. Each participant was given the opportunity to opt out, and patients who did not opt-out were considered to have provided tacit consent for study participation. The study protocol was reviewed and approved by the Ethics Committee of the Sapporo University School of Medicine (IRB:332–150).

## Results

### Patient characteristics

A total of 108 patients with IBD (56 with UC and 52 with CD) were included in the study. The baseline characteristics of the included patients are presented in Table 1. A total of 483 plasma samples were collected, of which 17 serum samples were available simultaneously.

**Table 1:** Baseline characteristics of the study population.

	CD (n = 52)	UC (n = 56)
Sex male (n, (%))	32 (61.5)	23 (41.1)
Age (y.o, median (IQR))	38 (27–50)	47 (33–57)
^a^Disease location	4 (7.7) /5 (9.6)	-
(L1/L2/L3/L4, n (%))	/43 (82.7) /0(0)	
^b^Disease extent	-	13 (23.2)/8 (14.3)
(E1/E2/E3, n (%))		/35 (62.5)
Plasma LRG	12.9 (9.2–16.9)	11.8 (8.6–15.8)
(μg/ml, median (IQR))		
Hemoglobin	13.6 (12–14.5)	13.2 (11.1–14.5)
(g/dL, median (IQR))		
Albumin (g/dL, median (IQR))	4 (3.8–4.3)	4 (3.7–4.3)
CRP (mg/dl, median (IQR))	0.1 (0.1–0.17)	0.1 (0.1–0.16)
^c^CDAI score (median (IQR))	137.5 (17.3–244.3)	-
pMayo score (median (IQR))	-	1 (0–3)
^d^SES-CD (median (IQR))	6 (3–11.5)	-
^e^UCEIS (median (IQR))	-	2.5 (1–5)
^f^MES (median (IQR))	-	1 (0–2)

Disease location and extent were assessed according to the Montreal classification.

^a^Crohn’s disease: L1, ileal; L2, colonic; L3, ileocolonic; L4, upper gastrointestinal involvement

^b^Ulcerative colitis: E1 = proctitis, E2 = left-sided colitis, E3 = pancolitis.

^c^CDAI: Crohn’s Disease Activity Index

^d^SES-CD: Simple Endoscopic Score for Crohn’s Disease

^e^UCEIS: Ulcerative Colitis Endoscopic Index of Severity

^f^MES: Mayo Endoscopic Subscore

### Correlation between serum and plasma LRG levels

**Fig 1:**
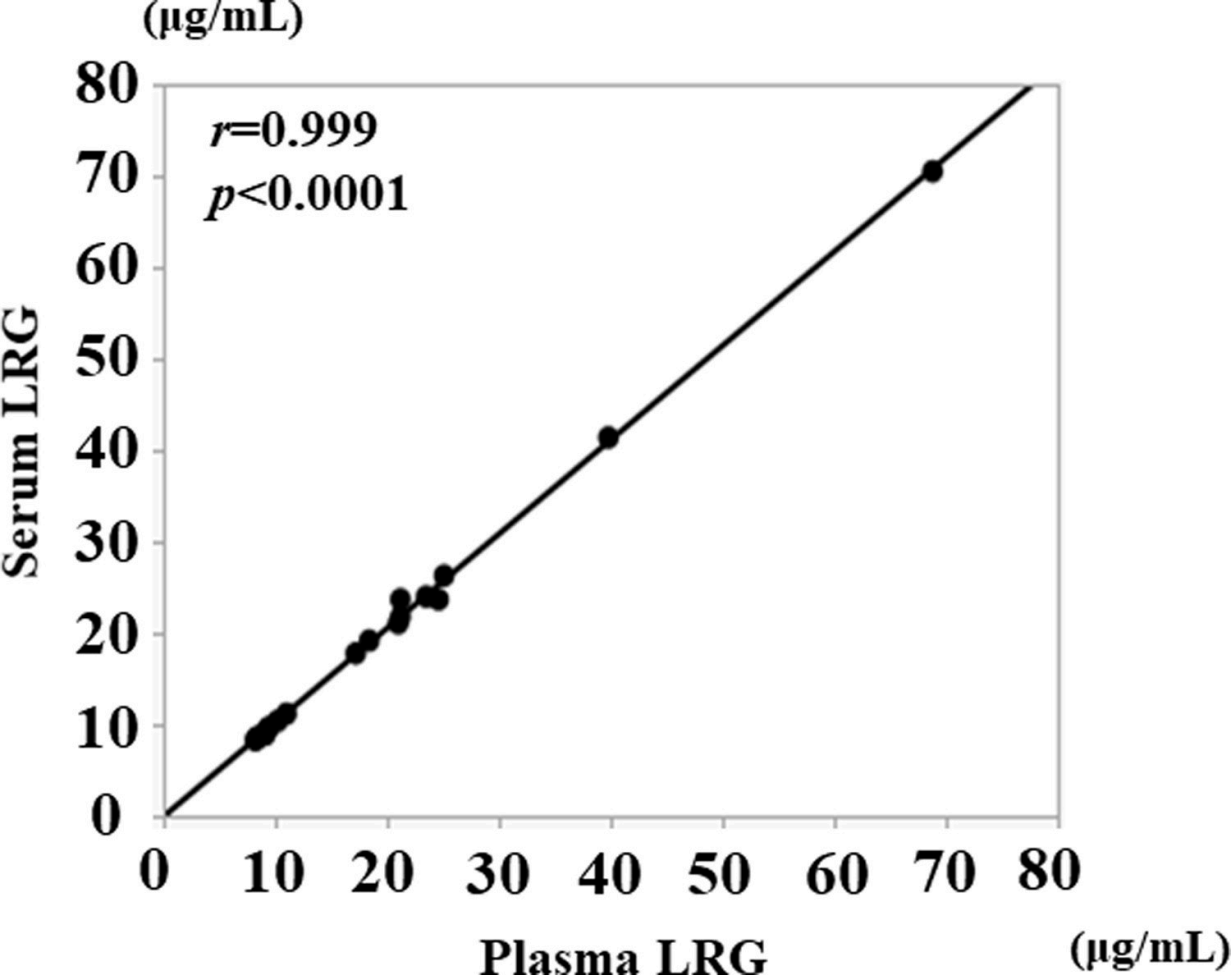
The correlation between serum and plasma LRG levels in patients with UC and CD.

We investigated the correlation between serum and plasma LRG levels using 17 paired samples obtained from patients with UC and CD. The serum and plasma LRG levels correlated well (*r* = 0.999, *p*<0.0001; Fig 1).

### Correlation with clinical and endoscopic disease activity

**Fig 2:**
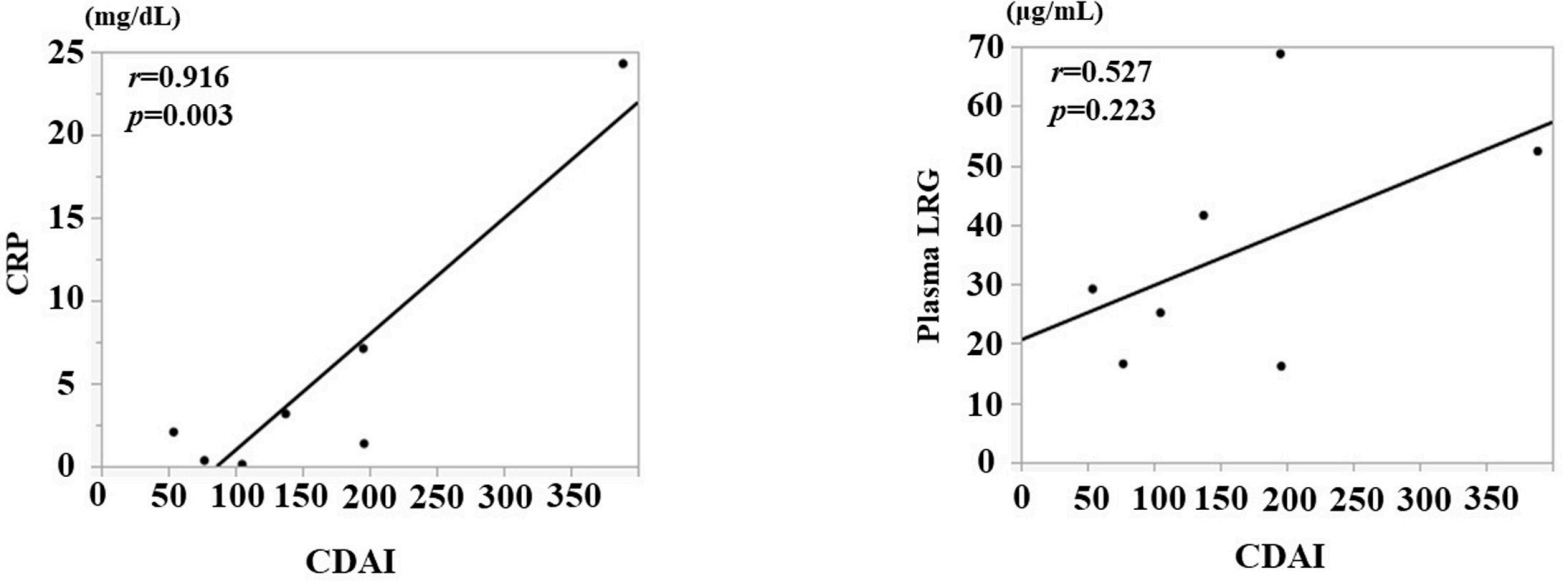
A. Correlation between CDAI score and CRP. B. Correlation between CDAI score and plasma LRG.

**Fig 3:**
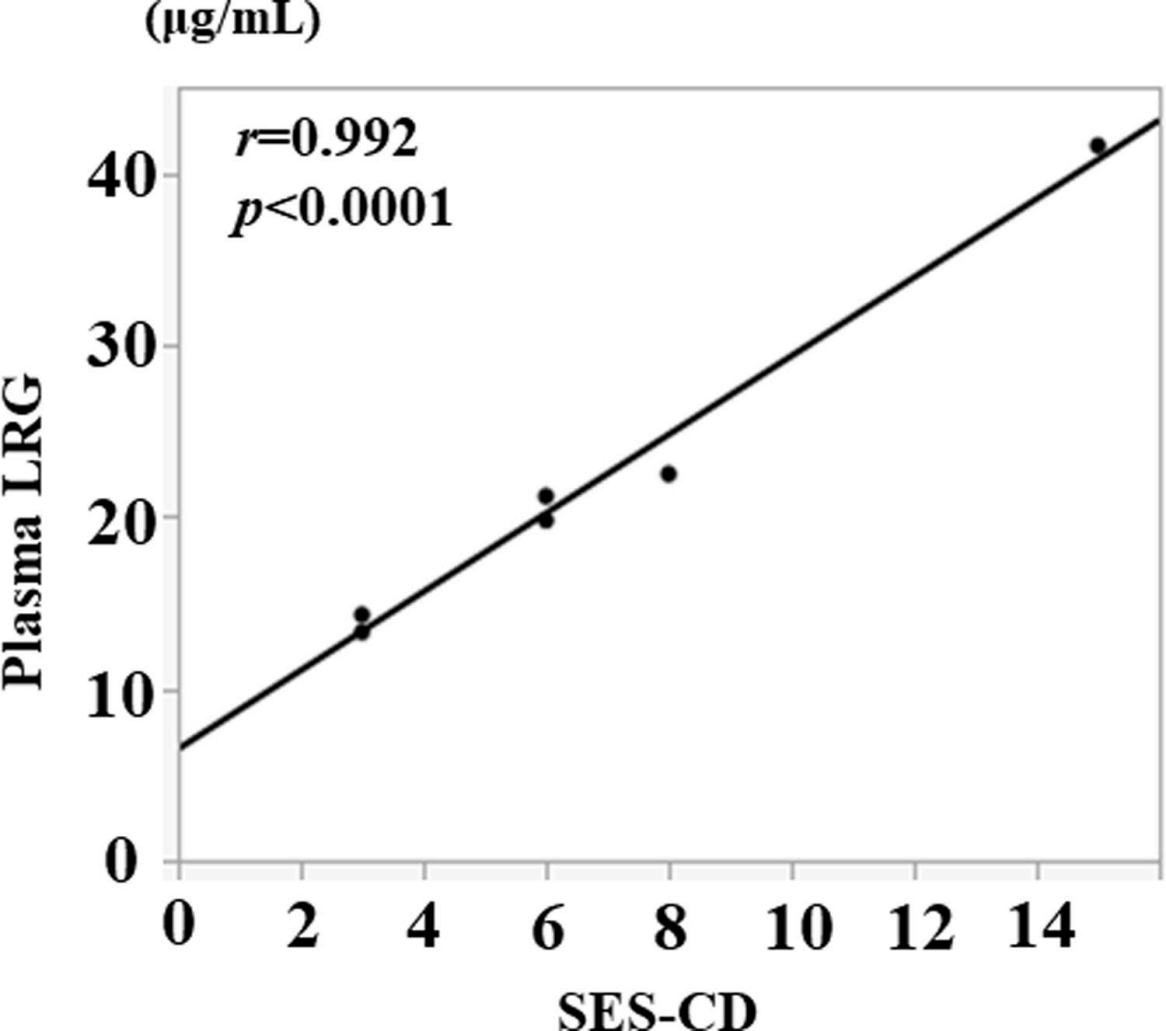
Correlation between SES-CD and plasma LRG.

The correlation between plasma LRG levels and clinical disease activity was also analyzed. The correlation between CDAI score and both CRP and plasma LRG levels was examined (Fig 2A and 2B). The correlation between CDAI score and CRP (*r* = 0.916, *p* = 0.003) was better than plasma LRG (*r* = 0.527, *p* = 0.223). However, plasma LRG levels were strongly correlated with SES-CD for endoscopic disease activity (*r* = 0.992, *p*<0.0001, Fig 3).

**Fig 4:**
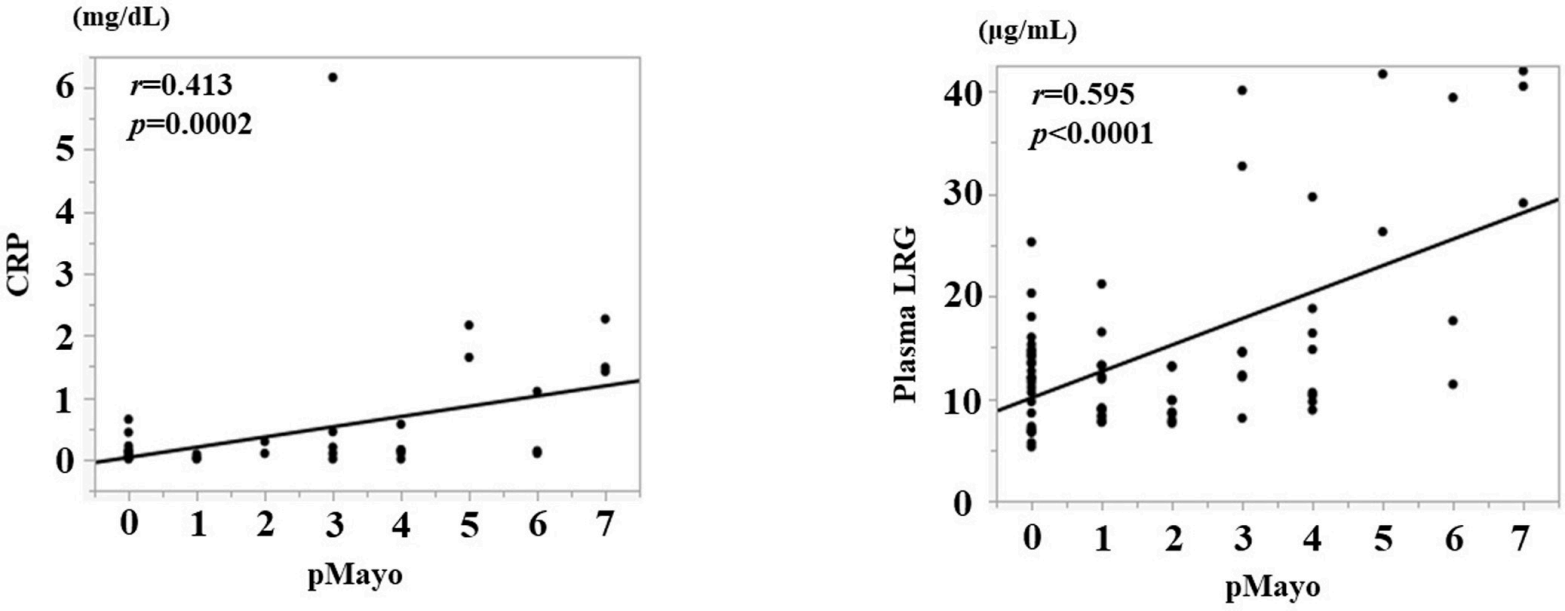
A. Correlation between pMayo score and CRP. B. Correlation between pMayo score and plasma LRG.

**Fig 5:**
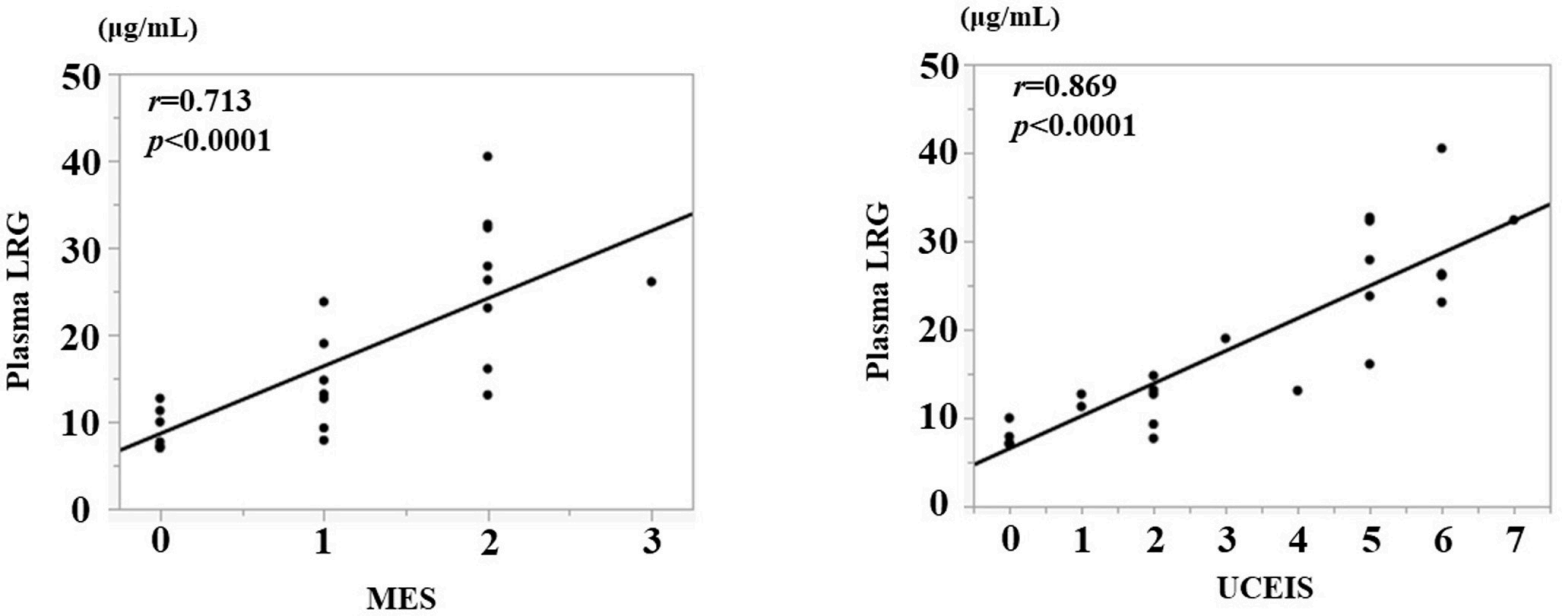
Correlation between plasma LRG and MES (A) and UCEIS (B).

Similarly, for UC, we analyzed the correlation between disease activity and CRP level, plasma LRG level, and endoscopic activity. Neither plasma LRG nor CRP showed a strong correlation with the pMayo score, but plasma LRG correlated better (LRG: *r* = 0.595, *p*<0.0001; CRP: *r* = 0.413, *p* = 0.0002; Fig 4A and 4B). Regarding the correlation with the endoscopic activity score, both MES and UCEIS showed a good correlation with the plasma LRG (*r* = 0.713, *p*<0.0001, *r* = 0.869, *p*<0.0001, Fig 5).

**Fig 6:**
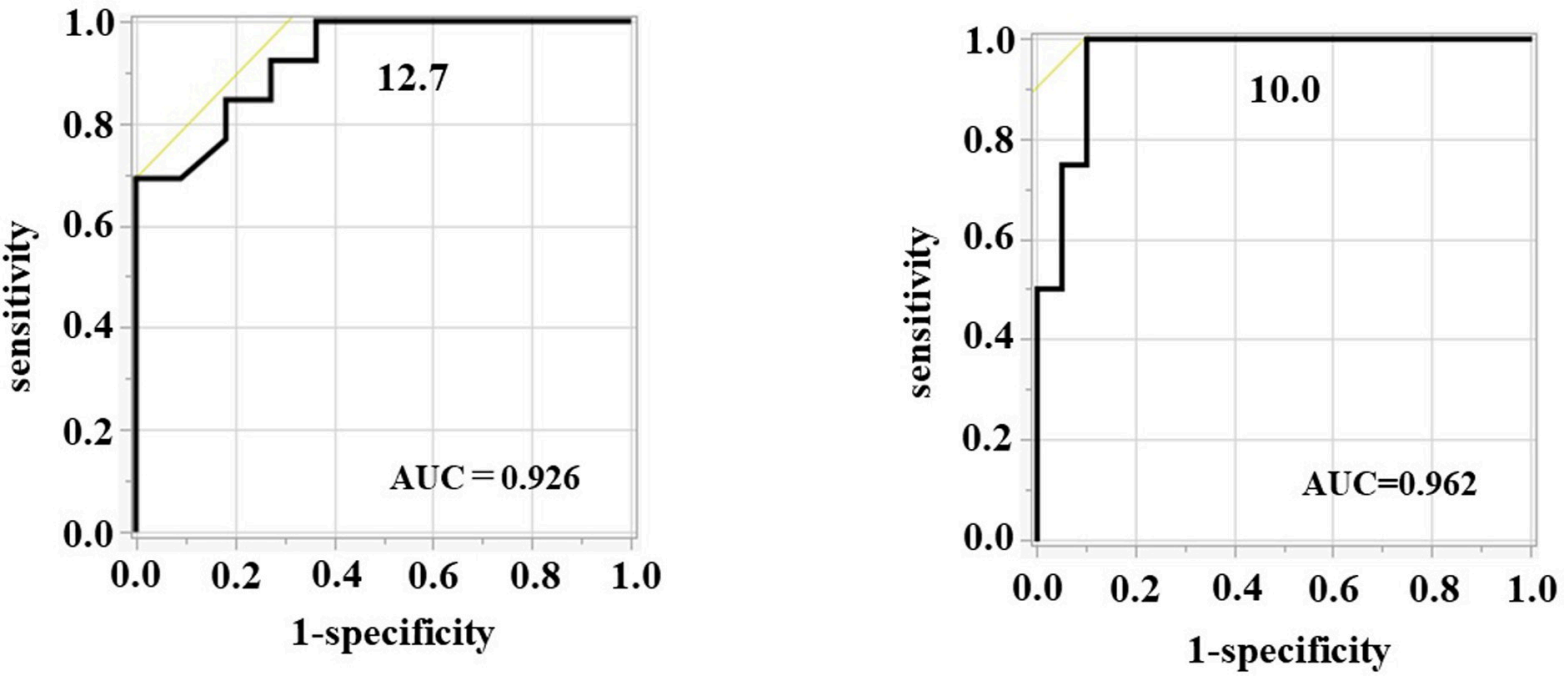
Diagnostic accuracy of plasma LRG for predicting endoscopic remission (MES ≤1 (A) and UCEIS = 0 (B)).

Receiver operating characteristic (ROC) curve analysis was performed to evaluate the diagnostic accuracy of plasma LRG level in predicting endoscopic remission in UC. Using MES, the area under the curve (AUC) for plasma LRG was 0.926 (95% confidence interval (CI):0.829–1.000). The optimal cutoff value for MES ≤1 was 12.7 μg/mL with a sensitivity of 69.2% and a specificity of 100.0% (Fig 6A).

Using the UCEIS, the AUC for plasma LRG was 0.962 (95%CI:0.891–1.000). The optimal cutoff value for UCEIS of = 0 was 10.0 μg/mL with a sensitivity of 100.0% and specificity of 90.0% (Fig 6B).

### Correlation with endoscopic activity in CRP-negative UC and CD

**Fig 7:**
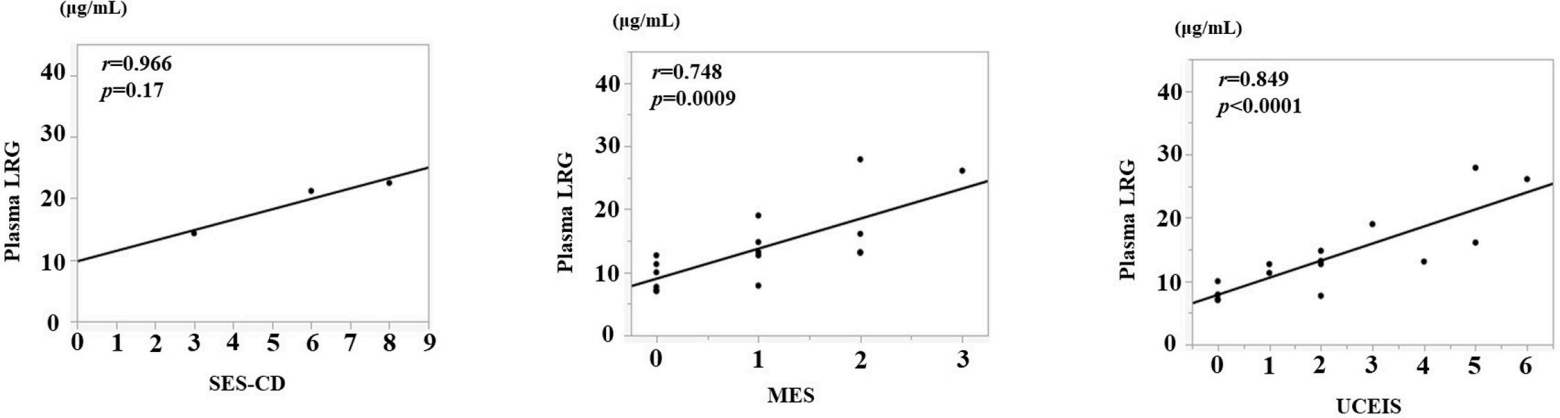
Correlation between plasma LRG and endoscopic disease activity (SES-CD (A), MES (B), and UCEIS (C)) in C-reactive protein (CRP) negative CD and UC patients.

We examined the correlation between endoscopic activity and plasma LRG levels in patients with CRP-negative UC or CD. Plasma LRG levels strongly correlated with SES-CD (*r* = 0.949, *p*<0.0138; Fig 7A). In CRP-negative patients with UC, a significant correlation was observed between plasma LRG levels and MES (*r* = 0.748, *p* = 0.0009; Fig 7B) and UCEIS (*r* = 0.849, *p*<0.0001; Fig 7C).

## Discussion

We found a strong correlation between serum and plasma LRG levels in patients with IBD. Plasma LRG levels were strongly correlated with endoscopic disease activity scores, even in patients with CRP-negative UC and CD. Measuring plasma LRG can shorten the incubation time at least 30 minutes compared to measuring serum LRG, which contributes to the rapid assessment of disease activity even in CRP-negative patients in daily practice. To the best of our knowledge, this is the first report to demonstrate the usefulness of plasma LRG levels in predicting endoscopic disease activity in patients with IBD.

LRG is a 50 kDa glycoprotein that contains repetitive sequences with leucine-rich motifs. In recent years, serum LRG has been gaining attention as a novel biomarker for patients in recent years [4, 5]. C-reactive protein (CRP) has also been used as a biomarker for inflammatory diseases, including IBD, for a long time; however, a previous study reported that 50% of CD patients showed negative CRP results even though they were endoscopically active [15]. LRG is induced by IL-6, IL-1β, TNF-α, and IL-22 [16]. Several reports have demonstrated a correlation between serum LRG levels and endoscopic disease activity in patients with UC and CD, even in negative CRP cases [17]. The use of anti-TNFα antibody preparations and JAK inhibitors may render negative CRP. In this cohort, 11 patients with UC showed negative CRP, contrary to the endoscopic disease activity (UCEIS >0). Of these patients, only one patient treated with 5-ASA + JAK inhibitor showed both negative CRP and negative plasma LRG. The remaining 10 cases, including each one patient treated with anti-TNFα antibody and JAK inhibitor + 5-ASA, showed negative CRP but positive plasma LRG. These data indicate that plasma LRG could detect endoscopic active but negative CRP cases receiving anti-TNFα antibody preparations and JAK inhibitors.

Although the NANOPIA LRG kit is applicable for both serum and plasma, no study has investigated the correlation between serum and plasma LRG levels and the clinical usefulness of plasma LRG in patients with IBD. In this study, we showed for the first time a strong correlation between serum and plasma LRG levels in patients with IBD. We also found that plasma LRG levels strongly correlated with endoscopic disease activity assessed using UCEIS, MES, and SES-CD, similar to previous reports using serum LRG [18]. These results suggest that plasma LRG could be an alternative to serum LRG to reflect endoscopic disease activity in patients with IBD.

We showed that the optimal cutoff value of plasma LRG for MES of ≤1 and UCEIS of = 0 was 12.7 μg/mL with a sensitivity of 100.0% and specificity of 69.2% and 10.0 μg/mL with a sensitivity of 100.0% and specificity of 90.0%, respectively. The threshold of serum LRG was set at 16.0 μg/mL to detect active IBD according to the manufacturer’s instructions; however, previous studies have reported that the optimal cutoff value for active IBD is much lower, approximately 13–14 μg/mL [6, 19, 20]. In the present study, the cutoff value was similar to that of previous reports in UC but was lower in CD.

This study had several limitations. First, the sample size is relatively small. In particular, few patients underwent colonoscopy, partly because some patients were reluctant to undergo endoscopy during the COVID-19 pandemic. In this study, the cutoff value of plasma LRG associated with endoscopically active CD was lower than that in previous data using serum LRG. The reason for this needs to be clarified. Generally, endoscopic scores are subjective rather than objective. Therefore, a more appropriate LRG cutoff level may be identified by assessing the degree of histological inflammation or its association with intestinal mucosal cytokine expression. Further studies with larger sample sizes are warranted to confirm the usefulness and optimal cutoff value of plasma LRG.

Second, this study cohort included several cases in which plasma LRG was measured multiple times in the same case, including some cases in which plasma LRG was measured before and after a treatment change. Although our data are heterogeneous, with different patient backgrounds and timing of measurements, we demonstrate the usefulness of plasma LRG in real-world clinical practice. Previous studies using serum LRG have shown its usefulness in prospective studies using the same agents [18]. Further prospective studies are needed to determine the factors associated with plasma LRG.

## Conclusion

In summary, the present study showed that plasma LRG data could be substituted for serum LRG data. LRG has been shown to correlate strongly with endoscopic scores such as SES-CD, MES, and UCEIS in patients with IBD. LRG may be a useful predictor of endoscopic findings.

## Supporting information

S1 Table Drugs administered to patients with negative CRP, contrary to the endoscopic activity (UCEIS >0). (DOCX)
