# Supplementary material for: Application of plasma alternative to serum for measuring leucine-rich α2-glycoprotein as a biomarker of inflammatory bowel disease
Source: PLoS One. 2023 Jun 23;18(6):e0286415. doi: 10.1371/journal.pone.0286415 (PMC10289387; doi:10.1371/journal.pone.0286415)
Supplement: S1 Table — (DOCX) [file pone.0286415.s001.docx]

**S1 Table**

**Drugs administered to patients with negative CRP, contrary to the endoscopic activity (UCEIS >0).**

| Drugs | Number of patients^a^ | |
| --- | --- | --- |
|  | CRP (-) / plasma LRG^b^ (+)  N=10 | CRP (-) / plasma LRG^b^ (-)  N=1 |
| 5-ASA ^c^/SASP ^d^ | 9 | 1 |
| IM ^e^ | 5 | 0 |
| CNI ^f^ | 1 | 0 |
| anti TNFα ^g^ | 1 | 1 |
| UST ^h^ | 1 | 0 |
| JAK ^i^ | 1 | 0 |

^a^ Including some duplicates.

^b^ Plasma LRG positivity was determined using the cutoff value of 10 μg/mL, calculated using the UCEIS in this study.

^c^ 5-ASA: 5-Aminosalicylic Acid, ^d^ SASP: Salazosulfapyridine, ^e^ IM: Immunomodulator, ^f^ CNI: Calcineurin inhibitor, ^g^ TNF: TNF-α inhibitor, ^h^ UST: Ustekinumab, ^i^ JAK: Janus kinase inhibitor.
